# Integrating High-Resolution Datasets to Target Mitigation Efforts for Improving Air Quality and Public Health in Urban Neighborhoods

**DOI:** 10.3390/ijerph13080790

**Published:** 2016-08-05

**Authors:** Vivek Shandas, Jackson Voelkel, Meenakshi Rao, Linda George

**Affiliations:** Toulan School of Urban Studies and Planning, Portland State University, 1825 SW Broadway, Portland, OR 97201, USA; jvoelkel@pdx.edu (J.V.); mrao@pdx.edu (M.R.); georgel@pdx.edu (L.G.)

**Keywords:** air pollution, dasymetric, urban, tree-planting

## Abstract

Reducing exposure to degraded air quality is essential for building healthy cities. Although air quality and population vary at fine spatial scales, current regulatory and public health frameworks assess human exposures using county- or city-scales. We build on a spatial analysis technique, dasymetric mapping, for allocating urban populations that, together with emerging fine-scale measurements of air pollution, addresses three objectives: (1) evaluate the role of spatial scale in estimating exposure; (2) identify urban communities that are disproportionately burdened by poor air quality; and (3) estimate reduction in mobile sources of pollutants due to local tree-planting efforts using nitrogen dioxide. Our results show a maximum value of 197% difference between cadastrally-informed dasymetric system (CIDS) and standard estimations of population exposure to degraded air quality for small spatial extent analyses, and a lack of substantial difference for large spatial extent analyses. These results provide the foundation for improving policies for managing air quality, and targeting mitigation efforts to address challenges of environmental justice.

## 1. Introduction

Unhealthy atmospheric conditions in urban environments are remarkably costly. Air pollutants from anthropogenic combustion processes pose a serious risk to public health and the environment [1], with the costs of exposure impacts estimated at over $220 billion annually in the US [2]; and an estimated 50 to 120 thousand premature deaths [3]. Recent evidence also suggests that long-term exposure to air pollution, especially respirable particles, can have profound impacts on cardiovascular risk factors, including artherosclerosis, hypertension, ischemic heart disease, and cardiovascular mortality [4,5]. Korek et al. (2015) [6], for example, found that long-term exposure at relatively low levels to nitrogen oxides (NOx and particulate matter (PM_10_)—two air pollutants created by combusting fossil fuels—from local traffic is significantly correlated to stroke. With the world’s population increasingly living and breathing in cities, mortality and morbidity rates due to air pollution are sure to rise, especially in rapidly industrializing countries. 

In the US, urban atmospheres are managed under a regulatory framework that assesses air quality at regional and national scales. However, current research in epidemiology [7,8], air quality [9,10,11,12], and environmental justice [13,14,15] clearly show that the issues of air pollution must be addressed at the neighborhood scale (≤1 km), because local hotspots that impact human vulnerability do not show up in regional analyses. Vulnerability, in this sense is the combined impact of exposure to degraded air quality and social conditions that can reduce the capacity to cope [16,17]. Additionally, regional assessments of human exposure, vulnerability, and coping with these adverse atmospheric threats ignore the role of municipal planning agencies, social drivers, and governance structures in exacerbating or ameliorating these threats [18,19], and limit the involvement of cities and neighborhood groups in improving urban health and well-being. For example, city managers and other decision-makers are expanding vegetation campaigns to improve air quality without a framework for understanding who is most impacted by degraded environmental conditions or, alternatively, those benefiting from these plantings. Thus, to substantially improve urban health and well-being, now and into the future, decision-makers need to simultaneously understand the distribution of air pollutants, identify the communities most exposed, and assess the effectiveness of mitigation strategies, such as tree-plantings, at the local scale.

Such an understanding about the drivers, impacts, and effective mitigation efforts, however, pose several challenges to a scientifically defensible and replicable approach. One formidable challenge is the heterogeneity of the urban environment, which results in high spatial variability of urban air pollution and, as a result limits accurate measurements of air pollutants at finer spatial scales [20,21]. Another significant limitation is that datasets for understanding who is being exposed to degraded air quality rely on data that assume uniform distribution of human populations at small spatial scales. For example, datasets on the distribution of human populations and socio-demographics are freely available through the US Census, and contain geographic extents that assume a distribution of people evenly across a spatial unit (i.e., block, block group, tract, county, etc.). Earlier studies examining linkages between human health and air pollutants, while increasingly commonplace, use these US Census datasets, often at the county [22,23], or tract [24] levels, which, while suitable for statewide or national estimates, fall short for characterizing local exposure. 

We posit that improving the resolution of exposure estimations requires three essential steps: (1) understanding the heterogeneous distribution of human populations in cities; (2) explicitly assessing the spatial variation of urban air pollutants; and (3) aligning the heterogeneous distribution of urban populations with a spatially commensurate highly-resolved air quality data [7,25,26]. The emergence of highly-resolved air quality monitoring techniques [27] and advances in spatial analysis offer a timely and promising approach to understanding those factors that mediate exposure to degraded environmental conditions. From the perspective of polices to improve human health and well-being, integrating high resolution datasets is a first step to identifying and targeting locations for the application of promising mitigation interventions.

In this study, we build on spatial analysis techniques for assessing air quality that can support city managers to better estimate the number of people exposed to degraded air quality, as well as to assess the extent to which one local mitigation strategy—tree-plantings—are effective in reducing exposure. Our approach begins by adapting and integrating two existing spatial analysis techniques to allocate urban population—cadastral and dasymetric mapping. We refer to our technique as a cadastrally-informed dasymetric system (CIDS): cadastral, because our maps employ high-resolution tax lot/parcel data which contain information on where residences are located and not located, such as on roadways, parks, factories, and other non-residential land uses; and dasymetric because we use disaggregated census-based population enumeration/aggregation geometries. Together—the residential tax lot to identify specific locations in our study area, and disaggregation of population estimates—provides an accurate distribution of the population. The population distribution is then combined with highly spatially-resolved nitrogen dioxide (NO_2_) data—an effective marker for anthropogenic combustion-related pollution—from a previously-developed observationally-based land-use regression model. These datasets help us to address three research questions: (1) What are differences in the exposure between household derived estimates of exposure to air pollution and those at different (or larger) spatial geographies? (2) Which locations and communities have the greatest exposure to air pollution, using NO_2_ as an indicator? (3) To what extent are neighborhood tree-planting campaigns effective in mitigating exposure to degraded air quality? 

Our research questions address several salient concerns about managing air quality in cities, including improving the spatial resolution of characterizing pollutants, the extent to which different demographic populations are disproportionally exposed to degraded air; and what short-term mitigation strategies show promise for improving local environmental conditions. To address these questions, we apply the CIDS approach to identify the location of communities most impacted by urban air pollution in the Portland Metro area. Next, we examine the extent to which Washington County’s (one of the three counties comprising our study area) re-vegetation efforts are directly impacting human health through the reduction of NO_2_ associated with tree canopy. Clean Water Services (CWS), the wastewater utility for Washington County, has invested in a major re-vegetation campaign since 2004, as part of a federal mandate. Now, over ten years after implementation, CWS has planted over 2 million trees, shrubs, and bulbs across 15,000 acres of land and several 100 of miles of riparian habitat, and anticipates another one million plantings in the coming years. Although our case study is in Portland Metro and Washington County, it is generally applicable to any part of the world, so long as comparable data exist. 

## 2. Materials and Methods 

### 2.1. Study Area 

Our study area is the Portland Metropolitan Area (PMA), a mid-size urban area covering 7967 km^2^, with a population of ~1.5 million (2013), located in the state of Oregon, in the Northwestern USA. It is situated at 45.52 N, 122.68 W, and has a temperate climate with relatively dry summers. The PMA is an ideal location for such a study for three reasons: (1) we have an abundance of highly resolved data for the region; (2) the tree canopy is diverse and, unlike many other cities, contains an extensive amount of tree canopy; and (3) community groups are actively in the process of expanding the tree canopy, although identifying locations for improving population health still requires further efforts. Approximately 29.4% of the PMA is under tree canopy. Mobile air pollutants, such as NO_2_, vary between summer and winter in Portland (summer and winter 2013 NO_2_ averages were 7.5 ppb and 11.4 ppb, respectively). Our study area includes Washington County, which is an area covering approximately 1882 km^2^, and is composed of both urban and rural areas. It is the fastest growing county in Oregon, with an estimated 500,000 people moving into the county by 2020. Approximately 26.7% of Washington County is under tree canopy.

### 2.2. Data Used and Analytical Process

We draw on several different datasets (Table 1) to address our research questions. Our overall methodology has three main steps: (i) using CIDS to develop a highly spatially-resolved population distribution map; (ii) developing a detailed exposure surface at three different spatial scales and evaluating differences—by exposure surface, we mean the distribution of NO_2_ and the total population who have an increased likelihood of exposure; and (iii) apply the CWS tree plantings at 5, 15, and 35 year growth projections, together with the reduction factor for the tree canopy, to assess the number of people benefiting from these plantings. Each of these steps is described in detail in the following sections. 

Using these datasets we were able to evaluate the level of exposure to an air pollutant (NO_2_) at three different scales. The first, using CIDS, assigns a population total at a tax-lot scale, and is described in further detail below. The second scale of analysis employs the US Census Block Group. The majority of Census Block Groups in the region contain between 600 and 3000 people, and vary in their geographic extent. We also used the US Census Tract, which contains between 1200 and 8000 people. The number of people in each of these geographic units was assessed in terms of potential exposure to the worst air quality and distance from the nearest highway. The results provide a means for evaluating the extent to which the characterization of geographic units affects potential exposure. 

#### 2.2.1. Cadastrally-Informed Dasyametric System (CIDS) for Population

Dasymetric mapping provides a means for higher accuracy areal population estimates through a process of disaggregation of enumeration units (e.g., United States Census Bureau Tracts or Block Groups) [28]. This disaggregation is necessary for high-accuracy analyses as it helps to combat the negative effects of the modifiable areal unit problem (MAUP) [29]. MAUP has been well documented in the literature on the exposure to environmental hazards as an issue occurring with non-uniform aggregation/enumeration units wherein results can be significantly different depending on which units of measurement are chosen for analysis [30,31,32]. By ”removing” population data from the constraints of Census geometries, we are able to examine them on a more human and realistic house-by-house level in contrast to an assumption of uniform distribution across a specified geography.

To construct a dasymetric map of the Portland Metro population, we use the tax lot (also known as a parcel or household) as the unit for allocating the population. In contrast to larger geographies available through the US Census (i.e., Census Block Group, Tract, County, etc.), where the population is assumed to be evenly distributed, the tax lot allocation can identify a more precise location and enumeration of the people residing at each tax lot. First, using 2015 tax lot data embedded with zoning and tax assessor information from the regional municipal government agency, Metro, we identify residential areas within each US Census block group (CBG) where the population can be allocated. We did this by filtering the 2015 tax lot dataset to create a new dataset containing only residential parcels. Using spatial analysis software (ArcMap Release 10.2, Redlands, California, ESRI Inc., Redlands, CA, USA), we extracted five categories of parcels using the embedded tax assessor data: Single Family Residential (SF), Multi-family Residential (MF), Rural, Agricultural, and Forested. Further processing of the filtered data resulted in a ‘Building Value’ measure that described whether the assessed monetary value of the building was zero (implying no home existed on the parcel) or a value greater than zero, suggesting a building was present. Any tax lots with a value of zero were removed from the list, along with those officially assessed as ”Vacant.“ Tax lots that spanned CBG boundaries were assigned to the CBG which included the centroid of the tax lot.

Since our tax assessor’s dataset for MF tax lots do not include the number of units, we developed a technique for attributing the number of people to specific units. To estimate the number of housing units in each MF we conducted a one-question phone survey that asked 60 randomly selected MF property managers how many units comprised their building. We developed an ordinary least squares (OLS) linear regression analysis using R statistical software (R Core Team, Version 2.15.3, Vienna Austria, R Foundation for Statistical Computing), to predict the number of housing units (based on the phone survey), with readily-available variables, including the land and building values (Equation (1)).

Predicted Housing Units MF = 3.261 + 1.573e**^−^**^5^ × LV + 8.858e**^−^**^6^ × BV (1)

where:

LV = Value in USD of land within tax lot

BV = Value in USD of buildings within tax lot

The results of the phone survey and our predicted housing units provided a high level of predictability (*r*^2^ = 0.97), which suggested that we can apply our results to all MF in the study region. OLS regression was employed as the initial exploratory analysis technique due to its simplicity and common usage—the high performance displayed left the authors satisfied that the method was sufficient and other more complex models did not need to be introduced. Using Equation (1), we calculated the predicted housing value for all MFs, and summarized for the CBG. The population to be assigned to each MF tax lot was then determined (Equation (2)), and used to develop a population density (people per square foot) estimate for both SF and MF polygons:

Population_Tax Lot, CBG_ = (Predicted Housing Unit_Taxlot_/Predicted Housing Unit _CBG_) × POP_CBG_ (2)

The population density was converted to a one square foot resolution grid wherein each pixel contained a single value representing population per square foot. The grid, or raster, dataset provided a computationally-efficient method for estimating population since the sum value of all pixels in the grid represented a population estimate in number of people. 

#### 2.2.2. Estimating Exposure Surfaces

##### Air Quality Assessments

Air quality was assessed using NO_2_ as a marker. NO_2_ is a ubiquitous urban air pollutant, produced by anthropogenic combustion processes. It is also one of the six US EPA criteria pollutants, and is the precursor to two other criteria pollutants, namely ozone and PM_2.5_ and, thus, serves as an excellent marker of air quality. The World Health Organization, in addition, recommends a threshold of NO_2_ below 40 μg/m^3^ [33]. In most urban areas in the US, combustion engines in automobiles are the biggest source of NO_2_. This NO_2_, emitted along traffic corridors is dispersed through turbulence and wind. Since it is a reactive gas, it also reacts to produce secondary pollutants (ozone) [34,35]. Studies find that NO_2_ levels are highest near the source, and then decay away from it, reaching background levels 200–400 m away [36]. 

We used the NO_2_ model developed by Rao et al. (2014) [37] (Equation (3)) for the Portland Metro area to assess air quality. Rao et al. were able to explain 80% of the variation in NO_2_ using a model that employed variables such as annual average daily traffic (AADT) and tree cover.

NO_2_ (i) = 7.7 + 1.1 × 10^−8^ × FWY_AADT_1200,i_ + 6.5 × 10^−4^ × MAJ_ART_500,i_ + 1.7 × 10^−3^ × ARTERIES_350,i_ + 1.8 × 10^−8^ × STREETS(POP)_800,i_ + 1.0 × 10^−3^ × RAILS_250,i_ − 1.0 × 10^−2^ × ELEVATION_i_ + 1.4 × 10^−5^ × (ELEVATION_i_)^2^ − 5.73 × 10^−6^ × TREES_400,i_ + 1.1 × 10^−4^ × X_DIST_i_ (3)

Adj R^2^ = 0.80, validation RMSE = 2.2.

where:
NO_2_(i)NO_2_ ppb, at site (i)FWY_AADT_1200,I_freeway (m) in 1200 m, weighted with AADTMAJ_ART_500,I_major arteries (m) in 500 mARTERIES_350,I_arteries (m) in 350 mSTREETS(POP)_800,I_streets (m) in 800 m, weighted by the population RAILS_250,I_railroads (m) in 250 m ELEVATION_i_elevation (ft)TREES_400,I_tree cover (m^2^) in 400 mX_DIST_i_distance from center of city (in m), along E-W axis

The model was created from direct observational data detected at 144 monitoring stations throughout the PMA, using passive Ogawa samplers. For more details on the field campaign and model development, please see Rao et al. (2014) [37]. As can be seen from the model, each hectare of tree cover within a 400 m radius of a site is associated with a 0.6 ppb reduction in NO_2_, holding all other variables constant. The model was applied to a 200 m point grid, which was then rasterized for faster calculations.

##### Integrating Population Estimates with Air Quality Assessments

We first identified all neighborhoods in Portland that had the most degraded air quality, which was identified as the worst quintile of nitrogen dioxide (13–24 ppb), based on the NO_2_ model. Next, to evaluate the effect of spatial scale of population estimates on potential exposure to NO_2_, we compared the air quality raster with the following population estimates: the tax lot using the CIDS approach, the US Census block group (as a population density raster), and the US Census track (as a population density raster) geographies. Population estimates for each geography were calculated using a zonal statistics technique (ArcMap Release 10.2, Redlands, California, ESRI Inc.). The result from our zonal statistics technique summarizes the population density value for all pixels that are within an area of interest (e.g., highway, highest concentrations of NO_2_, etc.). By comparing across the three geographies using the NO_2_ raster’s 80th percentile as a specific area of interest, we can estimate the number of people who are potentially exposed to the largest concentrations of NO_2_. 

In addition to looking at the potential neighborhood-level exposure, we further leveraged the CIDS population distribution to identify residences within 75 m and 150 m of regional highways (e.g., high traffic corridors). Current epidemiological research suggests that living in proximity to traffic corridors can create a disproportionate risk from traffic related pollution, including increasing incidence of asthma [38,39]; lung function deficit [40]; and cardiovascular problems [41]. Correctly identifying the population and demographics of people residing in pollution hot-spots such as traffic corridors can inform city-level policies on zoning (example, locating elementary schools >200 m away from freeways). Further, given the long history of neighborhood involvement in the study area, we also identified the neighborhoods in the PMA that lie in the highest quintile of NO_2_. Information about neighborhood NO_2_ can potentially engage and empower citizens with the worst NO_2_ levels to address the issue by implementing local mitigation strategies. Though the worst quintile of NO_2_ in our study area is highly associated with high traffic areas, the 75 m and 150 m areas of interest are drastically smaller and represent those locations where populations have been shown to have highest levels of health impacts.

#### 2.2.3. Case Study: Assessing the Mitigation of NO_2_ by CWS Tree Plantings

Washington County’s Clean Water Services, over the last decade, has planted trees at 162 planting sites across Washington County, Oregon. A typical planting site is small, covering an average of 0.02 km^2^. Although the main purpose of the plantings was to improve water quality, the small-scale nature of each planting makes it ideal to assess the potential of local mitigation strategies in improving air quality. CWS had a spatially explicit dataset with locations and extent of canopy-based restoration sites going back to 2004.

Since tree planting occurs when they are saplings we used existing literature [42] to model canopy growth associated with plantings at 5, 15, at 35 year intervals. Growth rates used for different functional types of plantings can be found in Table 2. Tree-related NO_2_ reduction on a site-by-site basis was assessed at these future intervals using Equation (3), and the number of people benefiting from a reduction in NO_2_ was estimated using the tax lot derived CIDS approach. Since we are unable to discern where population growth will occur in Washington County in the coming decades, our approach kept the population fixed at the 2010 levels over the entire 35 year period, which makes our estimates conservative in terms of the number of people experiencing benefits from the canopy campaigns. We note that, as population grows, the NO_2_ will also increase, and ours are likely conservative estimates of populations who are likely to be exposed to degraded air pollution. 

## 3. Results 

We divide our results into three sections, the first two of which describe the outcome of the CIDS approach, including the difference between our approach and those used in other estimates of potential exposure, and the role of tree plantings in reducing exposure. The final section addresses the implications of our study and caveats in applying these techniques to other cities and regions. 

### 3.1. Assessing Population Density and Potential Exposure Using CIDS

**Figure 1:**
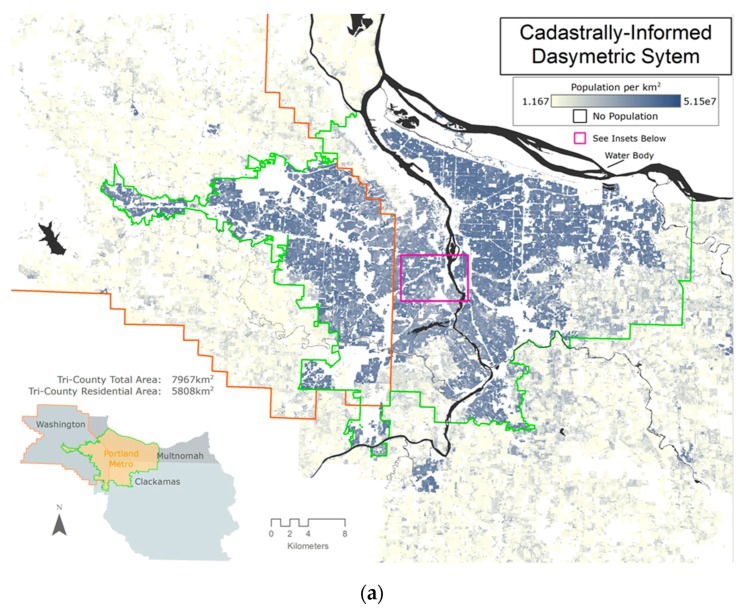

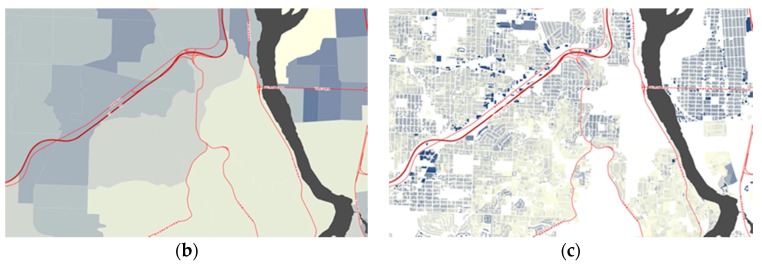
(**a**) (**Above**) the cadastrally-informed dasymetric map of the Portland Metropolitan region; (**b**) (**Left**) choropleth map of population density (population of Census Block Group divided by area of Census Block Group); and (**c**) (**Right**) dasymetric map of population density.

Dasymetric maps show promise in assessing exposure and risk because they provide a highly-resolved description about the underlying population density by using ancillary data for disaggregating population estimates, while remaining consistent with the census genealogy [43]. Arguably, accurate population density estimates are essential in assessing potential population exposure to the highly spatially variable pollutants, such as NO_2_, that are very common in urban areas. Our CIDS approach combined dasymetric allocation techniques with high-resolution cadastral land use and ownership data, to develop a highly spatially resolved (1 ft, 0.305 m) map of population density in the PMA (Figure 1a).

Our results reveal that applying the CIDS approach for allocating urban populations in the PMA changes the geographic extent of possible exposure locations. For example, the CIDS indicates that the total population of the PMA is distributed across 2159 km^2^, while methods of uniform population distribution at the county-scale would allocate the population across the entire tri-county area of 7967 km^2^. As a result, the CIDS approach would distribute population over just a quarter of the tri-county area (27%), while leaving 73% of the tri-county area non-residential. Similarly, for Washington County, CIDS allocates the population to 602 km^2^ as compared to the county area of 1882 km^2^, leaving 68% of the entire area of the county’s total land area unpopulated. 

A visual description between the two approaches provides additional support for moving toward a tax lot-based approach to likely exposure estimates via population density (Figure 1b,c). If, for example, the population is evenly distributed across a CBG that is adjacent to a source of air pollution (e.g., a high traffic corridor), then everybody in that CBG is assumed to be exposed to that source of pollution. Since a CBG can range from a few square kilometers in highly populated areas to hundreds of square miles in rural areas, assuming uniform exposure, given the spatial variability of urban air pollution, is inaccurate. In addition, identifying MF tax lots that are highly affected by air pollution can also help to target mitigation efforts, since such practices are costly and often unfeasible at large geographies.

We can expect, then, that potential exposure estimates based on methods, like CIDS, which account for where people actually live relative to sources of pollution, will provide greater accuracy for identifying populations most impacted by degraded air quality. To that end, we estimated the number of people exposed to air pollution (i) using NO_2_ as a marker for anthropogenic combustion-related pollution; (ii) the distance from freeways as a marker of traffic-related air pollution (high traffic corridor) using three geographic scales—tax lot (using our CIDS approach), US Census Block Groups (CBGs) (as a population density raster dataset), and US Census Tract (as a population density raster dataset). 

#### 3.1.1. Estimating Population—NO_2_ Worst Quintile

**Figure 2:**
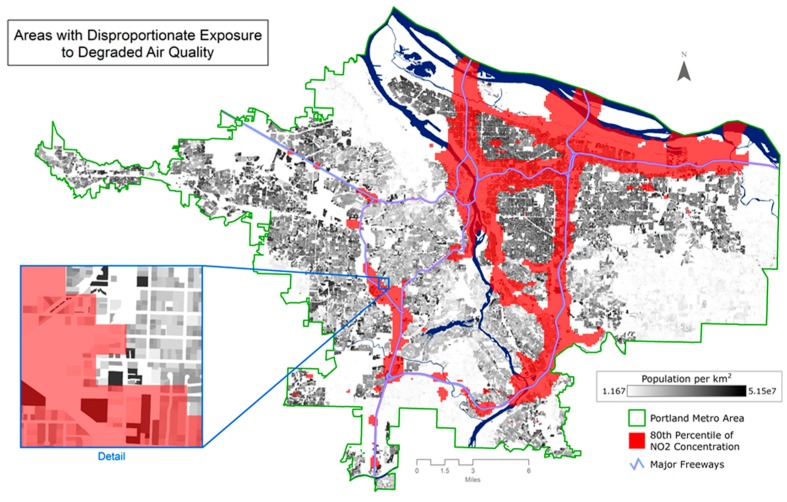
Distribution of the highest concentrations of NO_2_ in areas with the greatest number of people.

Based on the CIDS and a highly-resolved NO_2_ surface [37] (Figure 2), we estimated that ~330,500 people (18% of the Portland Metro population) are exposed to the worst quintile of NO_2_ pollution (13–24 ppb). Census Geography rasters were measured in the same manner by overlaying the area of interest over a rasterized choropleth of population density for both CBG and Census Tracts and calculating population values for only those specific pixels. Assuming the standard uniform population distribution over a CBG, the estimate of the exposed population is ~326,700, or about a 1% underestimate compared to the CIDS, while using census tract scale population data results in an estimate of 329,700 people exposed to the worst quintile of NO_2_, an estimate comparable to the CIDS (Table 3). Similarly, when examining the number of people exposed to the worst decile of NO_2_ (15–24 ppb), CIDS estimates 177,440 people, CBGs estimate 179,700 people, a 1% overestimate compared to CIDS, while the census tract-level population estimates result in a 2% overestimation compared to CIDS.

#### 3.1.2. Estimating Population—Highway Corridors

While the estimates with the CBGs and tracts may not substantially differ from the CIDS approach, in part because they are derived from the same data, an analysis of exposure from high traffic corridors, or highways, suggests an orders of magnitude difference in exposure. Based on existing literature, populations living near highway corridors are disproportionately at risk for asthma and other respiratory problems [21,38,44,45]. Using the CIDS and a highway corridor buffer of 75 m, we find that 12,700 people are at risk of traffic-induced respiratory problems. CBGs overestimate the population at risk by 169%, and tracts overestimate by 197% (Table 3). The CIDS in 150 m corridors, estimates 42,000 people at risk of traffic-induced respiratory problems, while CBGs overestimate the population at risk by 52%, and tracts overestimate by 66% (Table 3).

### 3.2. To What Extent Are Neighborhood Tree Planting Campaigns Effective in Mitigating Exposure to Degraded Air Quality?

**Figure 3:**
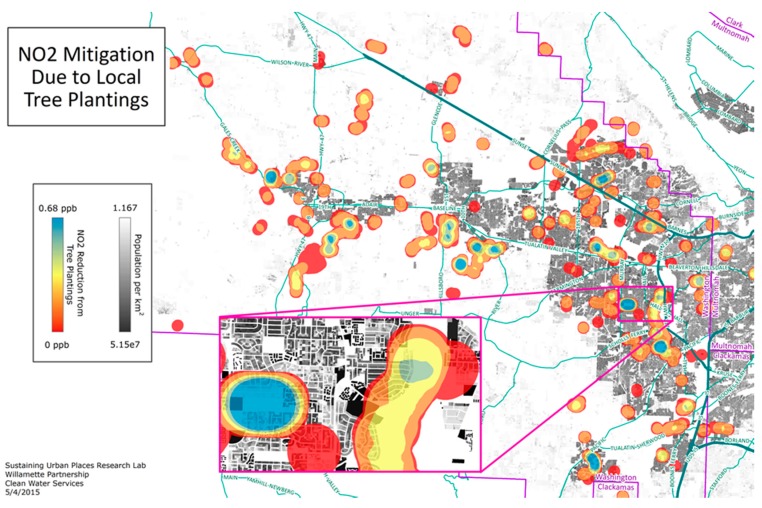
NO_2_ reduction associated with Clean Water Service (CWS) planting sites (35 year projection).

Many cities and neighborhoods are undertaking tree plantings, to beautify neighborhoods as well as to mitigate air pollution and urban heat. As part of an effort to understand the extent to which these local mitigation measures can improve degraded air quality, we evaluated the contribution to local air quality improvement within Washington County. Based on the results of an earlier study (Rao et al., 2014) [37], we determined that each of the planted sites could potentially be associated with a reduction in NO_2_ for up to 400 m from the site. Our results describe the number of people that are affected by the CWS tree plantings based on the canopy cover at 35 year maturity (Figure 3). Using the CIDS, and maintaining 2010 population, we find that ~142,300 Washington County residents will experience a non-zero reduction in NO_2_. We further estimated that ~9600 Washington County residents would experience a 2%–9% reduction in NO_2_ five years after planting, increasing to ~11,800 residents 15 years after planting, and ~20,500 would benefit from a 2%–9% reduction in NO_2_ 35 years after planting. The small size of a typical planting site and the small spatial extent over which tree canopy is seen to affect NO_2_ concentrations highlight the need for highly spatially resolved population, pollutant, and canopy data.

These results suggest that even in cases where an organization may not have directly considered the benefit of tree plantings to the immediately-adjacent populations, our CIDS approach provides a means for accounting for those who directly benefit. In targeting future tree plantings—a general program that is gaining increasingly popularity among municipalities across the globe—knowing how many people may benefit can be considered a direct measure of success. Certainly, decision-makers, planners, and public health advocates can assess the extent to which specific mitigation efforts has the greatest benefit to the largest number of people.

## 4. Discussion

The difference in population allocation strategies between US Census and CIDS alone provides one argument for the consideration of highly resolved population density estimations. Although only minor differences were detected between the CBG and tract estimates of population, and the CIDS, our analysis suggests considerable variation in potential exposure in the highway corridors. We observe that the smaller unit for assessing exposure, the greater the difference in estimates from CBG and tract estimates. The overestimation of exposure by CBGs and tracts as compared to the CIDS when estimating population within 75 m and 100 m of highways can be explained by the population allocation strategies used in these methods. Since the CBG and tract assume that the populations are evenly distributed, and also non-residential areas, those who are not directly adjacent to highways, are also counted as being exposed to degraded air quality. If, for example, a CBG (or tract) is adjacent to a highway, yet has a highly distributed population, all of the people in that CBG will be considered to have equal exposure which, in most cases, will not be accurate since distances greater than 150 m from the highway may not experience the same level of exposure as those immediately adjacent, that is, less than 150 m. When applying a tax lot-derived exposure estimate only those living adjacent to the highway (in this case 75, and 150 m) will be counted as potentially exposed. We posit that such differences are not trivial because they provide an accurate description of who is impacted by mobile sources of air pollution, and offer mitigation opportunities for targeting area where long-term exposure can lead to detrimental impacts on human health and well-being. 

Our results suggest a need to examine three inter-connected dimensions for reducing potential exposure to degraded air quality. First, characterizing the distribution of communities in urban landscapes requires greater scrutiny. One of the primary sources for population information in the United States is the Census, which is required by the constitution to be conducted every ten years. The U.S. Census has created a hierarchy of geographies—smallest is the block—with the population as the only consistent feature within each of the increasingly larger areas. Assumed within each geographic extent is the uniform distribution of individuals, which as our CIDs approach illustrates is not effective in understanding or characterizing environmental injustices. Moreover, our approach to identify MFR required developing an algorithm based on land values, and validating through individual phone calls. While time-intensive, such approaches are currently the only way to develop population distribution estimates for a heterogeneous land use mix. Needed are population distributions that take into account the land uses throughout a region. Several countries, including China, Germany, and England, have national land use datasets, which provides the foundation for applying our CIDS approach to other cities with nationally consistent land use systems. 

Second, the field of exposure studies is undergoing a renaissance, with new journals, conferences, and projects emerging throughout the world. Essential for advancing the field of exposure studies is empirically derived environmental quality information. The present study has the advantage of building on earlier assessments of NO_2_ concentrations, which are generally not available at our resolution for most cities and regions. By identifying and integrating places in the region with the worst air quality, and containing the most numbers of people, we can more accurately describe ‘hot spots’ for potential exposure. One limitation is that we only used one air pollutant (NO_2_), and a more comprehensive approach would identify particulate matter and/or black carbon, which have far-reaching consequences on human health. The emergence of several off-the-shelf air quality measurements systems offer several opportunities for integrating high-resolution description of environmental quality with the distribution of populations. Our approach provides an effective approach to addressing social vulnerability within the field of exposure studies. 

Finally, environmental justice literature is replete with description of communities that are disproportionately burdened with harmful air pollutants, yet missing from these studies are examples of approaches for identifying effective mitigation strategies. We attempted to use a modeling approach to describe the implication of a tree planting strategy to improve degraded air quality. By combining a spatially-explicit description of tree planting efforts with estimations for future vegetation growth, we created a model for evaluating the beneficiaries of such campaigns. As a result, rather than opportunistic application of urban canopy campaigns, our approach can enable natural resource managers, urban planners, and public health practitioners to strategically identify locations where air quality improvements are needed. While empirical validation of our assumptions for air quality improvements are needed, this approach offers a timely and systematic evaluation framework for committing resources to canopy campaigns. 

### Implications for Urban Health and Well-Being

Regulatory frameworks for addressing air pollution at the national and regional levels may obscure, or worse, completely miss, those most impacted by poor air. By integrating highly-resolved air quality data with residential locations, we identify locations, such as near freeways, where communities are likely experiencing harmful health outcomes. While our analysis does not directly assess exposure—rather, we use ambient concentrations of NO_2_ as a measure—a first step is to develop accurate descriptions about who and where degraded air quality is most likely to create adverse human health impacts. 

In the PMA we can expect, based on findings in the US and other developed nations, that populations exposed to the most degraded air quality may also lack the resources to combat the health impacts of air pollution, as they tend to have lower median household income and are more likely to be transient [46,47,48,49]. To that end, our results suggest a need for engaging both city planners and community groups in improving local air quality. The management of air quality in the US occurs at the regional or state jurisdictions and local decision-makers are not immediately charged with addressing degraded environmental conditions. Moreover, local decision-makers often lack access to data at the necessary spatial resolution to make effective decisions. Nevertheless, many cities are looking to improve local air quality through using climate action plans [50,51] and receiving help from federal agencies to pursue such efforts (see for example: US Environmental Protection Agency’s report entitled, “Air, Climate, and Energy Strategic Research Action Plan, 2012–2016”). 

Urban air pollution typically consists of highly reactive and highly spatially variable pollutants such as NO_2_, ozone, and fine particulate matter. While national interest in addressing environmental justice is growing the current state of the science needs a greater focus on integrating highly-resolved datasets. The Environmental Protection Agency, for example, has recently released an EJScreen™ for “environmental justice mapping and screening…that provides…a nationally consistent dataset and approach for combining environmental and demographic indicators,” which continues to rely on CBG, tract, and larger geographies for identifying specific communities at risk from degraded air quality. While such tools are helpful for national or state level assessments of environmental justice, local communities often require greater specificity (and resolution) for taking mitigation actions. 

New techniques that combine observational data with modeling—such as Land Use Regression (LUR) and Congestion Mitigation and Air Quality (CMAQ)—are emerging, and have the potential to provide spatially-explicit data for assessing finer, local-scale air pollution. However, without highly-resolved descriptions of the distribution of the urban population, we cannot identify those most exposed to degraded air quality and, hence, have little impact on the management of environmental determinants of human health. Given the ready availability of land use tax lot data and increasingly resolved air quality data, our approach offers a relatively seamless and accurate diagnostic approach for identifying locations where mitigation of air pollution needs immediate and sustained attention. 

In addition, at the neighborhood level, community groups are also highly engaged with improving local air quality. Groups in California, New York, Colorado, and other US states are working directly with state and local health officials to begin assessing the implications of improving neighborhood air quality. One notable example is a group in Oregon called Neighbors for Clean Air, which has mobilized community members, non-profit organizations, the scientific community, and government agencies in improving air quality for neighborhoods in Portland, Oregon [52]. Having spatially-resolved data can be essential to empowering local groups to take action, since many current policies for managing air quality reside at state and national levels.

## 5. Conclusions 

The design of cities that support human habitation requires at once the capacity to know those areas that are currently burdened with disproportionate environmental pollution, and understanding the role of short and long-term mitigation efforts. One of the main objectives of the present paper is to evaluate the differences in potential exposure among three different geographies. We developed a novel technique that integrates existing, highly-resolved air quality data with highly-resolved distribution of populations within one metropolitan area. We found that high-resolution descriptions of air quality and population distributions offer many advantages to describing the number of people impacted by degraded environmental conditions, with greater differences when examining specific areas, versus entire metropolitan regions. We also evaluated the extent to which tree-planting efforts can improve air quality for communities. By examining areas where these tree-planting efforts can improve air quality, we also provide a systematic approach to evaluating the beneficiaries of such campaigns. 

The results support an emerging area of exposure science, which is beginning to provide epidemiological evidence about human health outcomes from degraded environmental conditions [53]. The exposure science literature provides further evidence that tree-plantings at the scale of the city block are effective in improving air quality [54,55,56]. While our CIDS approach does not describe actual exposure—which itself is mediated through complex mechanisms of travel behavior and other activities—it does offer a first step to understanding how humans are potentially impacted by ambient air pollution. 

We also note that several municipalities are pursing green infrastructure projects as a means for mitigating air pollution. A recent example in the Seattle area, describes a coalition that is developing green infrastructure projects in the Lower Duamish—already a highly-polluted and lower-income population—that aim to improve air quality. Countless other examples illustrate community involvement in tree-planting efforts, many of which aim to improve the social and environmental conditions of historically marginalized neighborhoods. Our analysis provides support for urban tree-planting projects by further explicating the spatial distribution of benefits and burdens that associate to these efforts. This study goes further, however, to also argue that high-resolution descriptions about the co-location of urban tree-planting efforts, densities, and socio-demographics, is essential for reducing the social and environmental inequities that befall those who have the least access to resources. 

Arguably, the need to improve the resolution of air quality data is consistent with developing spatially-explicit approach to address mitigation strategies. From the perspective of human health and well-being, developing approaches that are directly relevant to existing planning and decision-making efforts is essential. Since air quality management in the US is largely the purview of federal air quality regulations, local planning bureaus have little authority for improving public health and safety in terms of ambient air quality. We posit that one challenge facing municipal decision-makers is identifying priority areas for addressing mitigation efforts. For example, since cities are constantly developing and modifying landscapes through permitting developments, identifying areas that are highly degraded, provides municipal managers with evidence for requiring mitigation efforts before allowing new (or re-) construction. Since land use planning occurs at the tax lot scale, our CIDS approach is one cost-effective and timely approach to assessing, and identifying, communities that are disproportionately impacted by poor air quality. These methods are one part of a broader agenda at enabling local communities to regulate pollutants, and mitigate their impact on those communities who have been historically burdened by degraded air. 

## Figures and Tables

**Table 1:** Description and source of data sets used in analysis.

Datasets	Description	Source	Year
Tax Lots	Polygon outlines of study area tax lots; fully attributed with Multnomah, Clackamas, and Washington County tax assessor data (including values and permissible types of building uses).	Regional Land Inventory System (RLIS)	2015
Freeways	Local major freeways which intersect the metropolitan area	RLIS	2015
Portland Neighborhoods	Portland Metropolitan Area-designated neighborhood boundaries	RLIS	2015
Park Boundaries	Polygon perimeter of all parks within the PMA	RLIS	2012
ACS	Census Block Groups with selected socio-demographic variables (specifically median household income).	US Census	2011
Census Block Groups	Topologies with select aggregate attributes including population. Vector data.	US Census	2010
Census Tracts	Topologies with select aggregate attributes including population. Vector data.	US Census	2010
Tree Planting	Discrete polygons with tree attributes including dominant species and extent of plantings.	Clean Water Services	2015
NO_2_ Model	High-resolution NO_2_ surface (200 m)	Adapted from Rao et al., 2014, with permission	2014

**Table 2:** Tree planting associated to functional type and increases in canopy cover (Washington County, OR, USA).

Location Functional Type	5 Years	15 Years	35 Years
Emergent Marsh	0%	0%	0%
Forested Wetland	25%	50%	85%
Oak Woodland	5%	25%	50%
Riparian Forest	25%	50%	85%
Riparian Forest Low Density	25%	45%	65%
Scrub shrub	25%	40%	75%
Scrub Shrub Low Density	20%	35%	55%
Upland Buffer	25%	50%	85%
Upland Forest	25%	50%	85%
Wet Prairie	0%	0%	0%

**Table 3:** Comparison of the total population exposed to degraded air quality using three geographic extents, and two distances from freeways.

	Number of People Exposed to NO_2_		Number of People Living Near Highways	
Exposure Unit	Worst Quintile (13–24 ppb)	Worst Decile (15–24 ppb)	75 m	150 m
**(CIDS) Tax Lot**	330,500	177,400	12,700	42,000
**CBG**	326,700 (−1%)	179,700 (+1%)	34,300 (+169%)	63,700 (+52%)
**TRACT**	329,700 (−0%)	181,400 (+2%)	37,800 (+197%)	69,600 (+66%)

CIDS: Cadastrally-Informed Dasymetric System; CBG: US Census Block Group; TRACT: US Census Tract.
